# Prenatal MRI Detection of Fetal Small Intestinal Volvulus: The Black‐and‐White and Coffee‐Bean Signs

**DOI:** 10.1002/ccr3.71787

**Published:** 2025-12-30

**Authors:** Miyu Nakamura, Hiroshi Sato, Kazuyo Kakui

**Affiliations:** ^1^ Department of Obstetrics and Gynecology Hyogo Prefectural Amagasaki General Medical Center Amagasaki Japan

**Keywords:** fetal intestinal volvulus, fetal MRI, neonatal surgery, prenatal diagnosis, small bowel obstruction

## Abstract

A rare case of fetal small intestinal volvulus was diagnosed prenatally using MRI, with characteristic black‐and‐white and coffee‐bean signs. Early diagnosis led to timely surgical intervention and a favorable outcome.

## 1 Case Description

**FIGURE 1:**
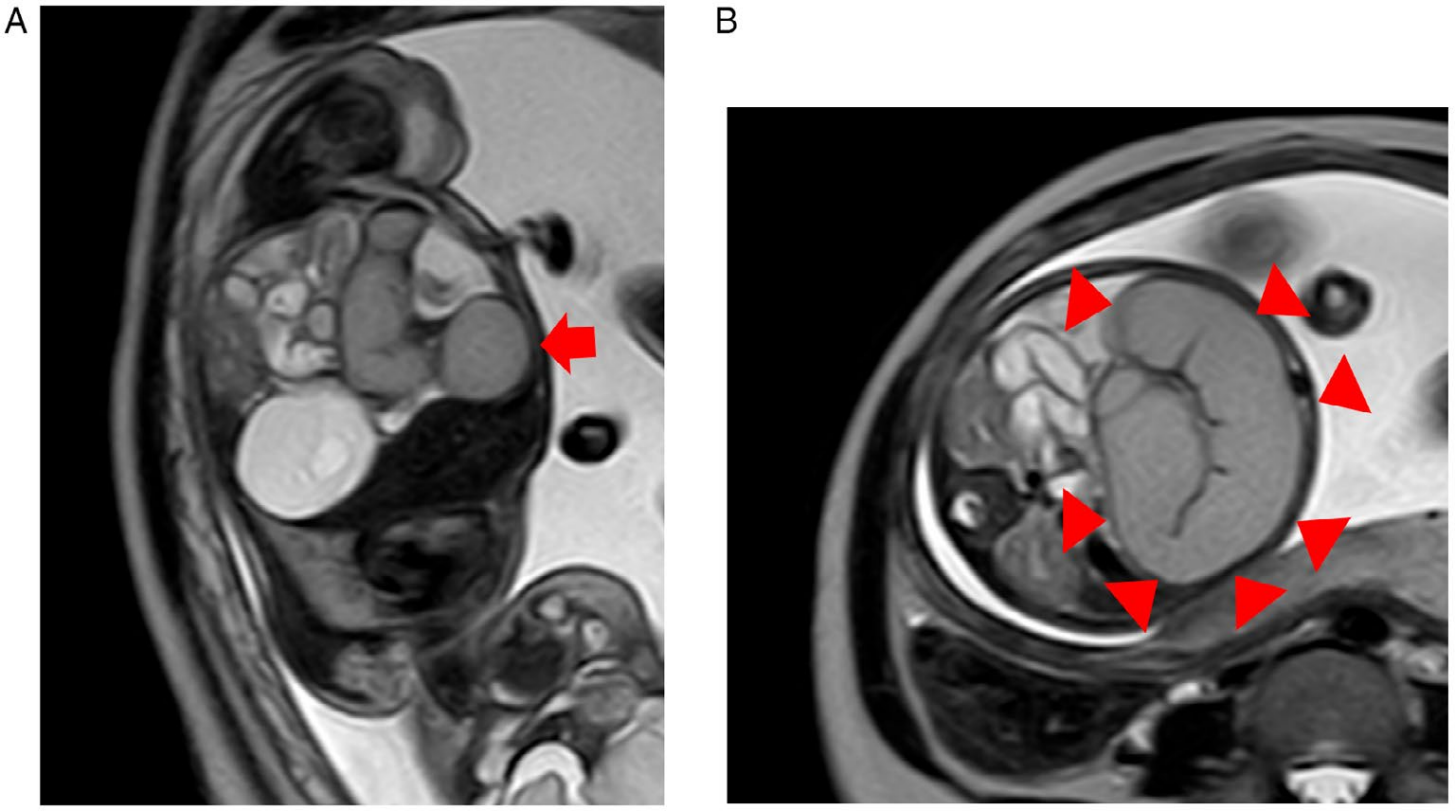
(A) Fetal MRI (T2‐weighted image) shows low signal intensity within the dilated small intestine (arrow), in contrast to adjacent normal bowel (“black‐and‐white sign”). (B) The twisted bowel loop (arrowhead) appears oval‐shaped with wall thickening—resembling a “coffee‐bean sign.”

**FIGURE 2:**
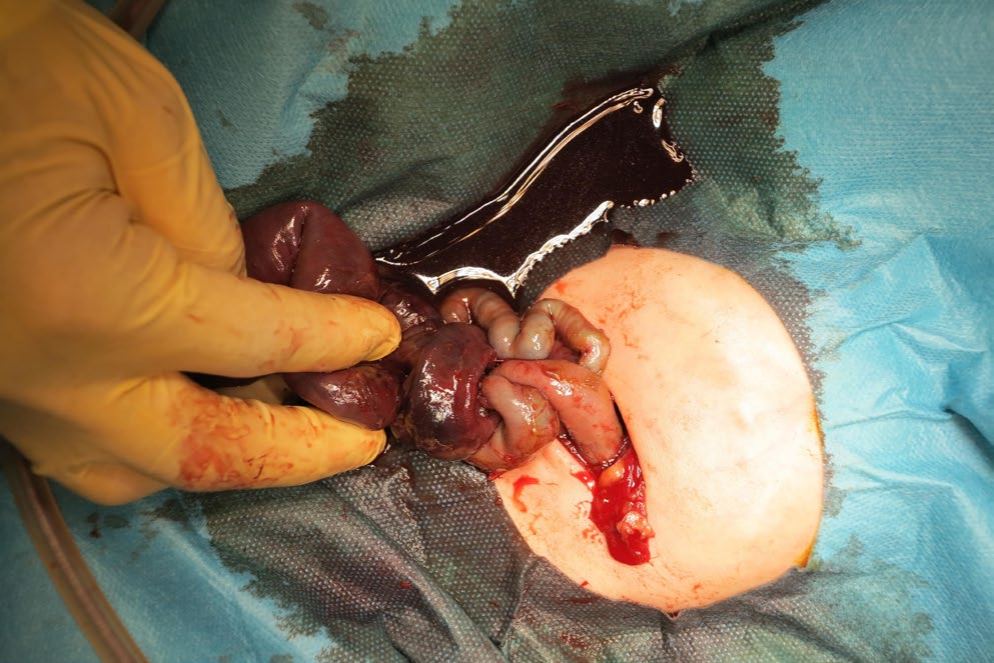
Postnatal laparotomy shows twisted and discolored small intestine with segmental perforation.

A 35‐year‐old Japanese woman presented with decreased fetal movement at 33 weeks. Ultrasound revealed fetal ascites and dilated intestinal tract. Because the cause of fetal ascites and bowel dilatation was uncertain on ultrasound, fetal MRI was performed to differentiate volvulus from intestinal atresia, meconium peritonitis, or bowel obstruction. Fetal MRI demonstrated characteristic findings: low T2 signal in dilated intestines (Figure 1A, “black‐and‐white sign”) and adjacent thickened oval loops suggesting torsion (Figure 1B, “coffee‐bean sign”). Emergency cesarean section was performed at 33 weeks, and the neonate underwent laparotomy 3 h after delivery. Laparotomy confirmed twisted necrotic small intestine (Figure 2). Segmental resection was performed, and the postoperative course was uneventful. The infant was discharged on postoperative day 50 and remained healthy at 3‐year follow‐up.

## 2 Answer

### 2.1 Diagnosis: Fetal Small Intestinal Volvulus

Intrauterine diagnosis of fetal small intestinal volvulus is increasing due to improved diagnostic techniques, although it remains rare [1]. Ultrasound is the main diagnostic tool, but not many cases show specific findings such as the whirlpool sign, and distinguishing it from gastrointestinal atresia can be difficult. However, when small bowel torsion occurs in utero, prognosis may be poor and forced delivery may be required, so accurate diagnosis is important. In recent years, MRI has been used for diagnosis of various fetal diseases, and as imaging time has shortened, diagnostic accuracy has improved. There are still few reports on fetal small intestinal volvulus diagnosed by MRI, but in addition to the whirlpool and coffee‐bean signs seen on ultrasound, the black‐and‐white sign reflecting ischemic changes has been described [2]. Fetal MRI should not be performed routinely in all cases with ascites, but it can be valuable when ultrasound findings are inconclusive or ischemic bowel change is suspected. Differential diagnoses include intestinal atresia, meconium peritonitis, and midgut malrotation. In volvulus, MRI typically shows the coffee‐bean sign and low T2 signal (“black‐and‐white sign”) indicating ischemia, which are not observed in simple atresia.

## Author Contributions

**Miyu Nakamura:** data curation, investigation, writing – original draft. **Hiroshi Sato:** conceptualization, formal analysis, investigation, writing – original draft, writing – review and editing. **Kazuyo Kakui:** supervision.

## Consent

Written informed consent was obtained from the patient for publication of this case report and accompanying images.

## Conflicts of Interest

The authors declare no conflicts of interest.

## Data Availability

The clinical data presented in this case report are not publicly available due to patient privacy, but further details are available from the corresponding author upon reasonable request and with appropriate ethical approval.
